# A Case Report of Generalized Non-pruritic Lichen Amyloidosis

**DOI:** 10.7759/cureus.39817

**Published:** 2023-06-01

**Authors:** Morgan A Rousseau, Stephanie A Valek, Rashid M Rashid

**Affiliations:** 1 Dermatology, University of Texas Health Science Center, Houston, USA; 2 Dermatology, Mosaic Dermatology, Houston, USA

**Keywords:** generalized rash, intense pruritus, amyloidosis, lichen amyloidosis, cutaneous hyperpigmentation

## Abstract

We evaluate the rare case of a patient who presented with generalized, non-pruritic lichen amyloidosis. There are three reported cases of generalized and non-pruritic lichen amyloidosis. The lichen amyloidosis subtype of primary localized cutaneous amyloidosis is characterized by keratinocyte-derived amyloid deposition in the papillary dermis, classically presenting as pruritic, hyperpigmented macules coalescing into plaques on the lower extremities. While the pathogenesis is likely multifactorial, chronic scratching has been proposed as an inciting factor. The patient’s type of lichen amyloidosis challenges the proposed etiology of chronic scratching leading to amyloid deposition.

## Introduction

Primary generalized, non-pruritic lichen amyloidosis (LA) is described only in three cases in the literature. Primary localized cutaneous amyloidosis is due to the deposition of extracellular amyloid in the skin without the involvement of internal organs. It is divided into three subtypes: lichen amyloidosis, macular amyloidosis, and nodular amyloidosis. LA is the most common subtype characterized by intensely pruritic hyperpigmented papules and plaques, most often on the lower extremities [1]. LA is thought to be due to scratching secondary to other pruritic conditions [1]. Few reports describe non-pruritic LA or generalized LA [2], but the combination of generalized, non-pruritic LA is rare [1,3,4].

## Case presentation

A 37-year-old female with no significant past medical history presented with a five-year history of darkening skin on the extremities. The patient described the hyperpigmented areas as non-pruritic and never scratched them. She denied excessive sun exposure, allergies, or a family history of similar skin symptoms. Her most recent blood work, including TSH and hemoglobin A1c, was normal. 

**Figure 1:**
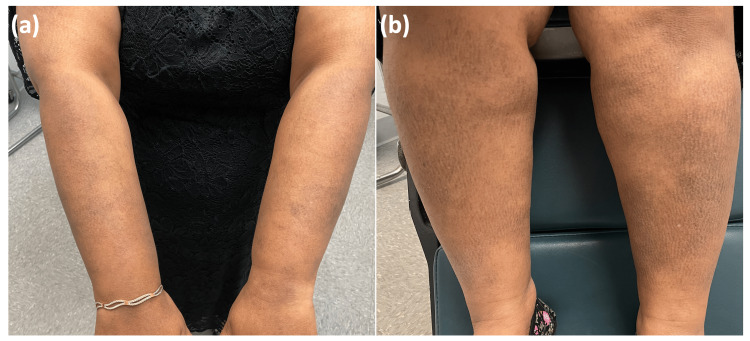
Anterior (a) distal upper extremities and (b) distal lower extremities with hyperpigmented palpable papules and macules

Physical exam was significant for subtle hyperpigmented palpable papules and macules on the upper and lower extremities coalescing into plaques in some areas (Figure 1). The pattern was geometric, with linear cut-offs at some areas' edges of normal-appearing skin.

**Figure 2:**
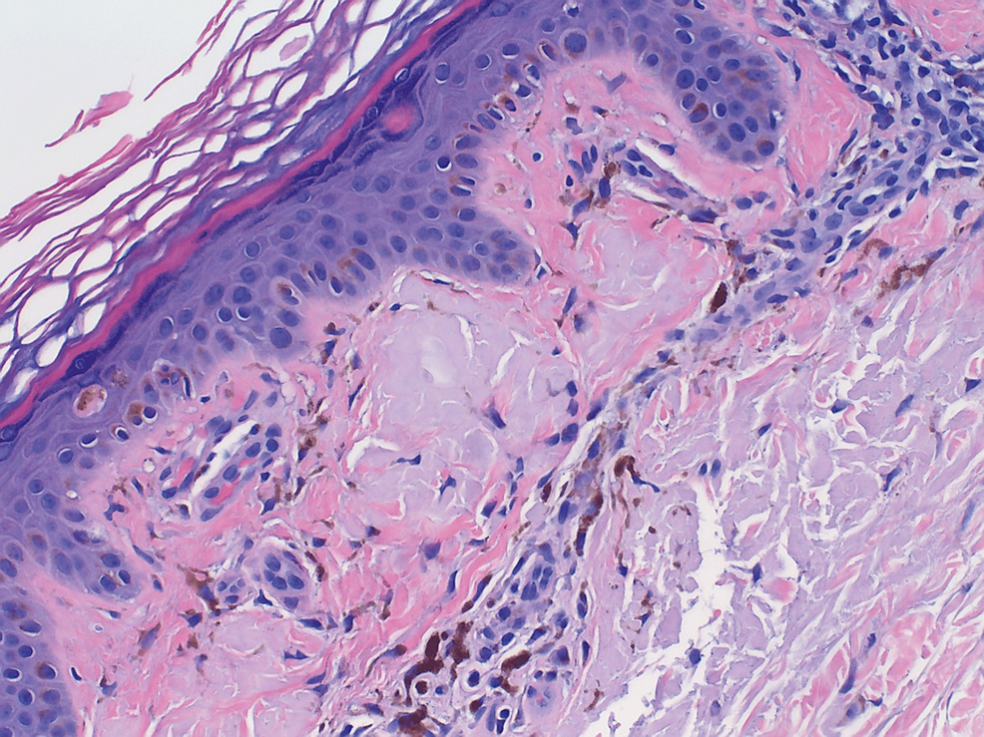
Biopsy stained with H&E at × 40 magnification demonstrating dull pink globules within the dermal papillae accompanied by melanophages, epidermal hyperplasia, and hyperkeratosis

Histopathology from a biopsy of the lesions showed dull pink globules within the dermal papillae accompanied by melanophages, epidermal hyperplasia, and hyperkeratosis (Figure 2), consistent with cutaneous amyloid. The histopathologic sample was confirmed, showing cutaneous amyloid with Congo red staining.

The patient's lesions were treated with triamcinolone 0.1% cream twice daily for two weeks without improvement. Treatment with hydroquinone 4% cream nightly for two weeks was also attempted, again without resolution of the lesions. The patient elected to forgo further treatment options at that time.

## Discussion

Degeneration of keratinocytes plays a vital role in the pathogenesis of LA, releasing cytokeratins that are deposited in the upper dermis as amyloid [5,6]. Apoptosis of keratinocytes may be triggered by chronic scratching, UV radiation, or viral infection [5]. While friction due to chronic scratching was traditionally considered the inciting etiology, this does not explain non-pruritic variants [1].

Lichen amyloidosis is also proposed to result from a sweat disturbance, where decreased sweating and skin dryness initiate amyloid deposition [7]. A separate study described a thermosensitive distribution of LA, sparing amyloid deposition in areas associated with the highest cutaneous temperatures [8]. However, a separate process might have led to our patient's generalized presentation, such as trauma due to routine shaving causing amyloid deposition. Alternatively, this variant might be a separate condition with similar histological findings.

Histologically, the characteristic homogenous amyloid deposits are confined to the papillary dermis and can be seen with stains such as Congo red or Crystal violet [9]. Other histological findings include irregular acanthosis, orthohyperkeratosis, and colloid bodies [2,9].

While most cases of LA are sporadic, up to 10% of cases are inherited in an autosomal dominant pattern with variable penetrance [9]. LA associated with Multiple Endocrine Neoplasia Type 2A has been well reported. In these patients, immunohistochemical staining is positive for keratin and negative for calcitonin [10].

In a case series of 15 patients with lichen amyloidosis, four cases (27%) were non-pruritic. Most cases were localized to the shins, with one patient presenting with generalized LA [2]. However, non-pruritic and generalized LA cases are rare, with only three reported cases [1,3,4].

The differential diagnosis for lichen amyloidosis includes lichen simplex chronicus, hypertrophic lichen planus, pretibial pruritic papular dermatitis, and pretibial myxedema [9]. Biopsy of these clinically similar conditions will not show amyloid deposits. Generalized LA shares feature with poikiloderma-like amyloidosis, which presents as generalized poikilodermatous papules that may be associated with blisters [9].

Treatment includes avoidance of scratching in addition to topical corticosteroids and keratolytic [4]. Other treatments have shown efficacy, including topical menthol, capsaicin, tacrolimus, amitriptyline, cepharanthine, colchicine, cyclophosphamide, cyclosporine combined with narrow-band UVB, retinoids, and laser or light therapy [9].

## Conclusions

While LA is often described as a localized, pruritic, hyperpigmented plaque, this is a rare case presenting as non-pruritic and partially generalized. Thermosensitivity, sweat disturbance, or epidermal trauma associated with shaving may provide more likely etiologies in this non-pruritic presentation. Further research is necessary to elucidate the pathophysiology of this unusual presentation of LA or characterize this presentation as a rare variant. Lastly, while this presentation of LA is non-pruritic, the associated hyperpigmentation can lead to significant patient distress, mainly due to the indolent nature of LA.
